# Transition milk or milk replacer powder as waste milk supplements to cold-stressed neonatal Holstein dairy calves: Effects on performance, feeding behavior, and health

**DOI:** 10.1371/journal.pone.0305227

**Published:** 2024-06-25

**Authors:** Borhan Moradi, Shahryar Kargar, Meysam Kanani, Morteza Nemati, Marzia Albenzio, Mariangela Caroprese, Ícaro Rainyer Rodrigues de Castro, Marcos Inácio Marcondes

**Affiliations:** 1 Department of Animal Science, School of Agriculture, Shiraz University, Shiraz, Iran; 2 Foudeh-Sepahan Agriculture and Animal Husbandry Center, Isfahan, Iran; 3 Department of Agriculture, Food, Natural Resources and Engineering (DAFNE), University of Foggia, Foggia, Italy; 4 Department of Animal Science, Universidade Federal de Viçosa (UFV), Av. Peter Henry Rolfs, s/n - Campus Universitário, Viçosa, Minas Gerais, Brazil; 5 Department of Animal Sciences, Washington State University, Pullman, Washington, United States of America; University of Guelph Ontario Agricultural College, CANADA

## Abstract

Young calves are more susceptible to cold than older animals due to their limited ability to regulate body temperature and lack of fat reserves and may have difficulty consuming the energy needed to cope with the cold by maintaining body temperature and meeting their metabolic needs, especially when fed constant levels of waste milk (WM) with less solids, which can be detrimental to health and future performance. An alternative to overcome this problem is increasing the milk’s solids content to the existing volume by using different sources [milk replacer powder (MR) or transition milk (TM)]. Thus, we aimed to evaluate the effects of increasing the total solids of WM via MR (WM+MR) or TM (WM+TM) on the performance, feeding behavior, and health-related variables of cold-stressed dairy calves during pre- and post-weaning. We hypothesized that feeding WM supplemented with MR or TM as potential liquid feed enhancers would improve milk dry matter and energy intake of the calves with a positive impact on body development and have no negative impact on feeding behavior and health. Additionally, we hypothesized that MR would not differ from TM. As a sample size calculation at 80% power using power analysis (PROC POWER) in SAS 9.4, a total of 51 Holstein-Friesian vigorous male calves [vigor score 21–27; 17 per treatment; 4-d old; body weight (BW) = 40.0 ± 0.63 kg (mean ± SD)] were selected, assigned randomly to treatments, and housed in individual pens in an outdoor barn. Irrespective of the type of treatment, all calves were fed 6 kg/d liquid feed from d 1 to d 53 of the experiment. In a step-down weaning program, calves received 0.5 kg liquid feed from d 54 to d 60. All calves were weaned on d 61 and remained in the study until d 101 as post-weaning evaluation. The calves had *ad libitum* access to starter feed and fresh drinking water across the experiment. Intake, growth, and behavior data were analyzed using a general linear mixed model and health data were analyzed using mixed logistic regression, mixed linear regression, and survival analysis models in SAS. We found that supplementation was responsible for a greater dry matter intake (DMI; *P* = 0.004), superior average BW (*P* = 0.037), and increased crude protein (CP; *P* = 0.001) and crude fat (CF; *P* = 0.001) intakes, with the most favorable outcomes observed for the WM+TM group when compared with WM+MR. Animals fed WM (control group; CON) showed a smaller average daily gain during the first 40-d of life (*P* = 0.026), showing slight changes during the whole period of evaluation when compared with the supplemented groups (SUP; WM+MR and WM+TM). No difference between MR- and TM-SUP groups, probability of having abnormal appearance (*P* = 0.032) and pneumonia occurrence (*P* = 0.022) was reduced in the SUP than in CON animals, with no effect on diarrhea among treatment groups (*P* = 0.461). Using milk supplements added to WM is an alternative to improve the intake, performance, and health of young calves under cold stress. Our findings showed that SUP animals outperformed the CON group in terms of DMI, average BW, and intake of CP and CF, with the TM-SUP group displaying the most favorable outcomes. Moreover, the SUP groups demonstrated reduced odds of experiencing abnormal appearance and pneumonia, highlighting the positive impact of supplementation on calf health.

## Introduction

The most challenging phase during young calves’ development is from birth to weaning. Studies on young calves showed that preweaning nutrition and environmental conditions affect biological health, physical functioning, and behavior [1–3], growth efficiency [4], and future performance of calves [5], which altogether will influence the economy of dairy farms. During this period, calves cope with physiological and environmental changes [6]. Therefore, proper nutrition and a dry, comfortable, clean, and non-stressful environment are essential for newborn calves to adapt effectively to their new surroundings [7,8]. The necessity of adaptation to a new environment is primarily due to the temperature variations experienced by the calf during delivery, as it transitions from the controlled conditions inside the cow to the outside world [9]. Therefore, providing adequate care and support is crucial to ensure the calf’s successful adjustment to its new environment.

Cold weather represents a major stressor for newborn calves as it affects the immune system and increases the susceptibility to enteric and respiratory problems [10]. Cold stress is a condition in which the environment temperature drops below the calves’ thermoneutral zone so that for thermoregulation, the rate of metabolic heat production increases [11]. In cold environments, the body undergoes physiological adaptation processes and hence, needs an increase in nutrient intake to regulate temperature by producing heat. Previous studies have demonstrated that livestock animals subjected to severe cold stress experience an elevated rectal temperature, muscle shivering, increased heart rate, and deeper breathing. Additionally, there is an observed increase in nutrient and energy requirements, all attributable to the heightened metabolic rate [12,13]. Also, it was recently reported that calves in the growing stage (6-mo-old) had an increased rectal temperature and heart rate as physiological responses in exposure to severe cold stress (-4.3 °C) than the threshold temperature (4.7 °C), as well as a decreased blood glucose level as a result of a reduction in dry matter intake [14]. The threshold temperature for experiencing cold stress is higher in neonatal calves compared to older ones. This is because neonatal calves have a greater ratio of surface area to body mass and underdeveloped heat conversion mechanisms, such as a low proportion of subcutaneous fat and thin skin, which result in increased body heat loss in cold conditions [15]. When the ambient temperature falls below the lower critical temperature (15 or 5°C for newborn calves younger than 21 days [16] or beyond 21 days of age [17]), the energy required to sustain core body temperature is provided either by the increased energy intake or from the increased metabolism of body tissue reserves and increased feed intake. As an instance, the results of a study showed lower daily weight gains as well as a required 32% more energy for maintenance for calves that were raised at -4 °C than those kept at 10 °C [18]. Furthermore, researchers found that the negative impacts of cold stress on newborn calf performance intensified because of immature cold defense mechanisms and high energy needs for BW [19]. These findings imply that providing more energy during the early life of calves via liquid or solid feed would reduce the adverse effects of cold stress on growth and health.

During the initial three days post-birth, calves predominantly rely on liquid feeds such as colostrum and transition milk (TM), for their nutritional requirements. Subsequently, starting from day four onward, various milk sources become part of their diet, such as saleable whole milk and milk replacer (MR), or non-saleable waste milk (WM) containing antibiotic or drug residues, lower-quality colostrum, and TM. This preweaning phase plays a pivotal role in calf growth and development, with proper nutrition and management significantly influencing their health, performance, and subsequent productivity within the dairy system [9,20,21]. The choice of milk feed type is often determined by factors such as availability, economic conditions, and farm practices [22]. As dairy farms increase in size, so does the volume of milk produced. With larger herds, there may be a proportional increase in the amount of non-saleable milk, or WM generated. This can be attributed to factors such as the higher incidence of mastitis in larger herds, leading to increased antibiotic treatments and subsequent withholding periods for milk, resulting in more milk being designated as waste, giving rise to the need for efficient utilization of this resource [23]. WM production can range from 22 to 62 kg per cow annually in the United States [24].

To address this, many dairy producers opt to use WM to feed their calves, turning a potential loss into an asset. Feeding WM is common practice in both America and Europe, with 87% and 33% of study farms, respectively, adopting this method [25]. It is also prevalent in Iran [26,27]. Furthermore, WM can expose young calves to numerous pathogens, and producers should pasteurize WM to inactivate microorganisms that would present a risk of transmitting infectious diseases [28–30]. The benefits of on-farm pasteurized milk include reducing diarrhea and pneumonia incidence and improved body weight gain [24]. While the total solids content remains stable in saleable whole milk, the DM content in WM experiences significant fluctuations based on the ratio of transitioning cows to treated cows. To ensure a balanced composition, supplementing WM with other types of milk is a possibility to increase its overall total solids content [31,32]. TM and MR are two types of liquid feed to increase the DM content of WM. The potential benefits of TM (yielded 2 to 6 d post-parturition [7]) on growth efficiency are documented [27]. While TM contains immunoglobulins (Igs), it’s worth noting that these Igs do not contribute to calves’ passive immunity after 24 hours post-birth. However, they still hold the potential to offer local immunity in the gut, which may help decrease susceptibility to enteric pathogens or diarrhea in young calves [27,33]. On the other hand, it is well shown that feeding MR solely instead of WM may slow down the small intestine development of newborn calves [34–36], while the small intestine is the main site of liquid feed digestion, it affects nutrient absorption and thereby calves’ growth and health in the early age of life [36]. These findings suggest that increasing the DM content of WM using TM or MR will probably be a good nutritional approach to optimize young calves’ growth and health.

During cold winters, many farmers increase the DM content of liquid feed offered to provide more energy without changing the total volume fed to the animals [37]. In addition, it has been seen that intensive feeding of liquid feed to neonatal calves increases nutrient intake, resulting in greater growth rate, BW, size at weaning, and animal performance reducing economic losses [11]. Moreover, calves born in winter consumed more starter feed compared to those born in other months, yet they achieved similar weight gains across all seasons [38,39]. These findings suggest that winter management strategies in dairy farming should focus on providing sufficient starter feed to calves born during this season, as they tend to consume more of it. Despite their increased feed intake, it’s crucial to note that their weight gains remain similar to those born in other seasons. Therefore, dairy farmers should ensure adequate feed supply during winter months to support the nutritional needs of calves and promote healthy growth. Thus, increasing milk DM would be an alternative to allow calves to overcome problems associated with cold temperatures [40]. However, the literature still lacks research regarding the specific effects and advantages of increasing the DM content of liquid feed, particularly milk, during cold winters for calves. Further research is needed to investigate the potential benefits, optimal DM levels, and long-term impacts on calf performance, health, and overall winter management strategies.

Several research studies have indicated a positive correlation between elevated DM content in milk, ranging from 17.6% to 20.4%, and enhanced calf performance [41–44]. Particularly, these studies primarily focused on performance metrics without delving into health and behavioral aspects. Moreover, it is worth highlighting that the application of this concept using TM and incorporating a feeding regimen involving WM, particularly in the context of calves subjected to cold ambient temperatures, remains an unexplored territory in the current body of knowledge.

Thus, we aimed to investigate the impact of increasing the total solids of WM via MR or TM theoretically to 14% of DM on the performance, feeding behavior, and health status of neonatal Holstein dairy calves under cold ambient temperatures. We hypothesized that using MR or TM as potential WM balancers would improve milk DM and energy intake of neonatal Holstein dairy calves under cold ambient temperatures with positive effects on growth performance and no effect on feeding behavior and health. Secondly, we hypothesized that MR would have the same benefits as TM when supplementing WM.

## Materials and methods

The present study was conducted from October 2019 to February 2020, at the Foudeh-Sepahan Agriculture and Animal Husbandry (Isfahan, Iran), when the mean ambient temperature was 4.2 °C ± 3.4 °C (mean ± SD). Following the daily official microclimatic reports of the nearest Meteorological Station at Isfahan International Airport (Isfahan, Iran), the average values for wind velocity, and maximum and minimum relative humidity were 2.4 m/s, 76.6%, and 31.3%, respectively. All procedures were done to comply with the Animal Care and Use Committee of Shiraz University (Shiraz, Iran; IACUC # 9731916) as suggested by the Iranian Council of Animal Care [45].

### Animals, treatment groups, and management

Based on an α of 0.05 and a power of 80%, the sample size was calculated at 17 calves per treatment group (51 calves in total). The 51 Holstein-Friesian young calves (n = 17 per treatment; 4 days of age; BW = 40.0 ± 0.63 kg; blood total protein = 6.55 ± 0.16 g/dL; dam parity = 2.5 ± 0.16; mean ± SD) were randomly housed in a naturally ventilated barn equipped with individual pens (2.9 m × 1.1 m × 1.8 m; length × width × height). Daily, manure was removed, and bedding was replaced with wheat straw with a 2-d interval to keep the pens visibly dry and clean.

Several criteria were used to select the calves in the current work. First, the herd veterinarian examined the health status of each calf at birth using the vigor scoring system [46]. Calves with good (score 21–22) or higher vigor [very good (score 23–25) or excellent (score 26–27)] scores were selected to enroll in the study. Calves exhibiting diarrhea, fever, physical disabilities, failure of suckling, and other health-related problems were not enrolled in the trial. Next, Holstein male calves with a birth weight between 35 to 45 kg along with a 24-h total circulatory protein level greater than 5.5 g/dL were balanced for body weight (BW), blood total protein, and dam parity. The animals were then assigned to individual pens (three calves daily; one calf/treatment per day) for 17 successive days.

Each calf received pasteurized colostrum (5.0 kg total; 60 °C for 90 min; Model IG-PLUS; Shirmack Pasteurizer, Shirmack Livestock Engineering Group, Isfahan, Iran; Brix value ≥ 22.0%) using a nipple bottle where 3.0 kg were fed within the first 2-h after birth followed by 2.0 kg feeding 6-h after that. During days 2 and 3 of life, each calf was provided with 4 kg pasteurized TM (60 °C for 90 min) split into two meals and fed in steel buckets (at 0900 and 1700 h). From the 4^th^ day onwards, the calves were individually fed with pasteurized **WM** [67.5 °C for 30 min; non-saleable milk containing antibiotic and/or drug residues; DM = 10.33% ± 0.85; mean ± SD (Table 1)] that was identified as the control group (**CON**) or pasteurized WM supplemented with either **MR** powder [WM+MR; Imperial; Novin Roshd Shahran Foudeh, Isfahan, Iran; containing 22% crude protein (**CP**) and 17% fat in the composition; DM = 14.26% ± 0.78; mean ± SD] or **TM** (WM+TM; DM = 13.94% ± 0.76; mean ± SD) as the supplemented groups (**SUP**). The liquid feeds were provided to the calves using steel buckets, 6.0 kg/d from d 1 to d 53, 5.0 kg on d 54, 4.0 kg/d from d 55 to d 56, 3.0 kg/d from d 57 to d 58, and 2.0 kg on d 59 through 2 meals of equal volume (at 0900 and 1700 h) and 0.5 kg (morning feeding) on d 60 of the study period. On the 61^st^ day of the trial, the calves were weaned and kept in the same individual pens throughout the 101 days. Daily, the waste milk collected on the farm was transported to a separate facility with a refrigerator and blender, which housed a bulk tank. After pasteurization, the milk was then provided to the calves. The temperature of the liquid feeds was set at 40 ± 1.0 °C for feeding to the calves, and the MR powder and pasteurized TM were added to WM in two separate bulk tanks before feeding to the calves. At this point, milk refusals were recorded. From d-1 through d-101 of the trial, the animals had unlimited access to starter feed and fresh water. Calf handlers and trial personnel were trained and aware of the treatment allocation at the different stages throughout the experiment.

**Table 1:** Average daily nutrient composition of waste milk (WM) and a blend of WM with milk replacer powder^1^ (WM+MR) or transition milk (WM+TM) as liquid feeds fed to newborn Holstein calves.

Chemical composition	Treatments		
	WM	WM+MR	WM+TM
DM, % of as fed	10.33 ± 0.85	14.26 ± 0.78	13.94 ± 0.76
CP, % of DM	23.13 ± 1.11	22.43 ± 0.66	31.45 ± 3.69
Fat, % of DM	24.65 ± 1.98	23.18 ± 1.28	25.47 ± 3.20
Lactose, % of DM	34.12 ± 2.96	34.08 ± 1.03	24.34 ± 2.47
ME, Mcal/kg of DM	5.02 ± 0.13	4.95 ± 0.07	5.20 ± 0.15
CP:ME ratio, g/Mcal	46.08 ± 0.22	45.31 ± 0.15	60.48 ± 0.19
pH	6.83 ± 0.15	6.49 ± 0.11	6.17 ± 0.27

^1^Milk replacer powder (Imperial; Novin Roshd Shahran Foudeh, Isfahan, Iran) contained skim milk, whey powder, whey protein concentrate, vegetable fat, vitamins (contained per kg of product: 25000 IU of vitamin A, 5000 IU of vitamin D3, 150 mg vitamin E, 8 mg vitamin B1, 10 mg vitamin B2, 6 mg vitamin B6, 300 mg vitamin C, 2 mg vitamin K, 300 mg choline chloride, and 200 mg biotin), minerals (contained per kg of product: 80 mg of Zn, 70 mg of Mn, 100 mg of Fe, 1 mg of I, 0.4 mg Se, and 0.6 mg Co), and saccharomyces cerevisiae (10 mg per kg of product). Milk replacer contained (on DM basis) 22% CP, 17% fat, 0.4% crude fiber, 8% ash, 0.9% calcium, and 0.6% phosphorous.

### Sampling and analyses

Pooled pasteurized liquid feeds were sampled daily (two samples/day; one sample at each feeding time), stored at 4 °C, and transferred to the Central Milk Testing Laboratory of the farm to quantify DM, CP, fat, and lactose concentrations using an infra-red analyzer (MilkoScan 134 BN; Foss Electric, Hillerød, Denmark). In addition, a portable pH meter (model AZ8685; AZ Instrument Corp., Taichung, Taiwan) was used to measure the pH of liquid feeds (Table 1). Lastly, the daily nutrient intake was calculated based on the diet composition.

24h following the first feeding of colostrum, blood samples were taken (jugular vein puncture) and collected in spray-coated silica tubes (BD Vacutainer, Franklin Lakes, NJ, USA) to measure the serum total protein using a clinical refractometer (Model ATA-2771; Atago Co. Ltd., Tokyo, Japan). The average serum total protein (± SD) was 6.70 ± 0.12, 6.53 ± 0.16, and 6.42 ± 0.15 g/dL for WM, WM+TM, and WM+MR treatment groups, respectively.

To determine the DM and nutrient composition, samples of starter feed (n = 11; pooled within trial period) and orts (n = 10 per calf; pooled by calf within treatment) were taken immediately before the morning feeding every 10-d during the experiment. A forced-air oven was used to determine the DM content by drying at 100 °C for 24 h, method 925.40 [47]. The samples were ground, sifted through a 1-mm screen in a Wiley mill (Ogawa Seiki Co., Ltd., Tokyo, Japan), and analyzed in triplicate for CP (Kjeltec 1030 Auto Analyzer, Tecator, Höganäs, Sweden; method 955.04 [47]), ether-extract (**EE**; method 920.39 [47]), ash (method 942.05 [47]), and neutral detergent fiber (**NDF**) using a heat-stable α-amylase (100 μL/0.5 g of sample) and sodium sulfite [48]. The non-fibrous carbohydrate (**NFC**) component was calculated as 100 –(CP + NDF + EE + Ash) (Table 2) [49].

**Table 2:** Ingredients, chemical composition (% of DM unless otherwise noted), and particle size distribution of the basal starter feed.

Item	Value
Ingredient composition	
Alfalfa hay	10.00
Wheat bran	7.10
Corn grain, ground	47.00
Barley grain, ground	14.10
Soybean meal	18.80
Calcium carbonate	1.20
Vitamin and mineral mixture^1^	0.57
Salt	0.47
Magnesium oxide	0.38
Bentonite	0.38
Chemical composition	
Dry matter (DM), % of as fed	91.92
Crude protein (CP)	19.10
Non-fibrous carbohydrate (NFC)^2^	52.83
Neutral detergent fiber (NDF)	19.21
Ether-extract (EE)	3.24
Ash	5.73
Calcium^3^	0.74
Phosphorous^3^	0.43
Metabolizable energy (ME),^3^ Mcal/kg of DM	3.01
CP:ME ratio (g/Mcal)	63.46
Particle size distribution (% of DM retained on sieves)	
4.75 mm	19.65
2.36 mm	15.35
1.18 mm	35.30
0.6 mm	20.01
Pan	9.69
pef_>2.36_^4^	0.35
pef_>1.18_^4^	0.70
pef_>0.6_^4^	0.90
peNDF_>2.36_^4^	6.71
peNDF_>1.18_^4^	13.50
peNDF_>0.6_^4^	17.32
Xgm,^5^ mm	1.80
SDgm,^6^ mm	1.54

^1^Contained per kilogram of the supplement: 975,000 IU of vitamin A, 750,000 IU of vitamin D, 1,800 IU of vitamin E, 143 g of Zn, 76 g of Mn, 48.6 g of Cu, 19.5 g of Se, 18.4 g of Fe, 8 g of Ca, and 1.3 g of Co.

^2^NFC = 100 - (CP + NDF + EE + Ash); calculated from NRC (2001).

^3^Calculated from NRC (2001).

^4^pef>2.36, 1.18, and 0.6 = physical effectiveness factor determined as the proportion of particles retained on 2 (4.75 and 2.36 mm), 3 (4.75, 2.36, and 1.18 mm), and 4 (4.75, 2.36, 1.18, and 0.6 mm) sieves; peNDF>2.36, 1.18, and 0.6 = physically effective NDF determined as NDF content of starter feed diets multiplied by pef>2.36, 1.18, and 0.6, respectively.

^5^Geometric mean particle size, calculated according to ASAE (1995) method S424.1.

^6^Geometric SD of particle size, calculated according to ASAE (1995) method S424.1.

Additional samples of the basal starter feed (n = 11; one sample every 10-d throughout the experiment) and individual refusals (n = 5; pooled by calf every 10-d over the trial period initiating on d 51 through d 101 of the trial period) were collected and sifted into five fractions using a 4-screen particle separator (4.75, 2.36, 1.18, and 0.6 mm; Model 120; Automatic Sieve Shaker, Techno Khak, Khavaran, Tehran, Iran) [50]. To determine the particle size distribution, 200 g of each sample (basal starter or orts) was placed on the top screen and the stack of sieves was shaken for about 10 min so that the distribution of materials remained without any changes [51] After sifting, the DM content of each separated fraction was measured by drying at 100 °C for 24 h using a forced-air oven (method 925.40) [47]. The physical effectiveness factor (pef) was calculated as the DM proportion of particles retained on sieves two (pef _> 2.36_), three (pef _> 1.18_), and four (pef _> 0.6_) [52]. The physically effective NDF on sieves two (peNDF _> 2.36_), three (peNDF _> 1.18_), and four (peNDF _> 0.6_) was calculated by multiplying the NDF concentration of the feed by the fraction of pef_>2.36_, pef_>1.18_, and pef_>0.6_, respectively. The geometric mean particle size of the starter feed diet (Table 2) was calculated according to method S424.1 [53].

### Feed intake and growth performance

The starter feed offered and refused (taken daily at 1000 h before delivery of fresh starter diet) were weighed using a calibrated electronic scale (model SF-400; Etminan Co., Tehran, Iran) to record the individual feed intake of each calf daily. Starter feed was offered at a rate that allowed at least 10% refusals; therefore, daily starter feed intake was adjusted as the calf aged. The total nutrient intake was computed as the intake of each nutrient originating from the liquid feeds plus the starter feed diet.

The calves were weighed at birth, d 1 of the trial, and every 10 days after that before the morning feeding using a calibrated electronic scale (Model WLC; Etemad Co., Tehran, Iran). The difference between BW taken at 10-d intervals divided by 10 was considered the average daily gain (ADG; kg of BW/d). To calculate the feed efficiency, the weight gain was divided by total DM intake (milk DM + starter feed DM) or total ME intake (milk ME + starter feed ME). Body frame size, including the heart girth (circumference of the chest), body barrel (circumference of the belly before morning feeding), withers height (distance from the base of the front feet to the withers), body length (distance between the points of shoulder and rump), hip height (distance from the base of the rear feet to hook bones), and hip width (distance between the points of hook bones) were measured using a tape at the initial (d 1) and 10-d intervals thereafter over the study.

### Sorting behavior and feeding activity

To find whether the calves have been sorting the starter feed diet for each particle size fraction, the sorting value was generated per calf/10-d starting on d 51 through d 101 of the trial. The ratio of actual intake to the expected intake for particles retained on each sieve was used to calculate the sorting activity [54]. The predicted intake of an individual fraction was computed as the total diet DM intake multiplied by the DM percentage of that fraction in the fed starter where values of = 100, <100, and >100% were considered as no sorting, sorting against, and sorting for each particle size, respectively.

The calves were monitored visually for feeding and chewing behaviors including eating, ruminating, resting, drinking, standing, lying, and non-nutritive oral behaviors (**NNOB**; when the animal licked any surface, tongue rolling, etc.) every 5 min for an 8-h period (between 1000 and 1800 h) during two separate phases, including three successive days pre-weaning (d 57–59 of the trial) and three successive days post-weaning (d 87–89 of the trial) [55]. One observation (at least) of eating activity occurring after at least 5 min without eating behavior was considered a period of eating. The number of bouts during the 8 hours was defined as meal frequency. The time duration from the start point of the first feeding event through an interval between events was regarded as meal length (min/meal) which was averaged for each calf. Inter-meal intervals (min) were calculated from the end of a feeding event to the beginning of the next and averaged for each calf. To determine the speed of eating (g starter feed DM/min), the total amount of starter feed DM consumed during the 8 hours was divided by time devoted to eating and averaged for each calf. The total amount of starter feed DM intake consumed during each meal was recorded as the meal size (g starter feed DM/meal). The rumination pattern was calculated similarly.

### Health

The health status of the calves was checked daily, during the milk-feeding period (d 1 to 61), wherein the appetite and desire to consume the liquid and starter feeds, along with the general appearance were evaluated [56]. General appearance scores were assigned on a 1-to-5 scale: 1 = normal and alert; 2 = ears drooped; 3 = head and ears drooped, dull eyes, slightly lethargic; 4 = head and ears drooped, dull eyes, lethargic; and 5 = severely lethargic [56]. The physical shape and consistency of fecal materials of individually penned calves were evaluated based on a 1-to-5 score, where 1 = normal; 2 = soft to loose; 3 = loose to watery; 4 = watery, mucous, and slightly bloody; and 5 = watery, mucous, and bloody, before the morning feeding daily [56]. The fecal score was categorized as the number of days with a fecal score ≥3, and the general appearance was categorized as the number of days with a general appearance score ≥2. These categories were denoted as days with abnormal fecal scores and general appearance, respectively [57–59]. All the calves were evaluated daily for pneumonia according to the UW Calf Health Chart, which assigns and sums the eye discharge score, nasal discharge score, ear tilt score, cough score, and temperature score [60]. However, a further diagnostic step was done by a proficient veterinarian with years of experience in diagnosing and treating calf diseases to ensure the accuracy of the pneumonia diagnosis. The rectal temperature was recorded using a digital thermometer (model CT20; EmsiG GmbH, Hamburg, Germany), where the temperature of 39.4 °C was regarded as a fever threshold, according to which the calves recording a higher temperature with a general abnormal appearance, fecal score, or cough were examined by the veterinarian, unaware of the treatments to confirm whether the animals were suffering from diarrhea or pneumonia. Whenever any calf was detected as diarrheic or pneumonic was treated according to standard procedures at the Foudeh-Sepahan Agriculture and Animal Husbandry (Isfahan, Iran). Calves with diarrhea orally received a water-based rehydration salt solution [ORS; containing 500 mg dextrose, 250 mg sodium chloride, and 250 mg sodium bicarbonate per g; 4 L/d (10 g ORS/L) per calf in 2 meals of equal volume (at 1200 and 2000 h)] for five consecutive days (Rooyan-e-Isfahan Co., Isfahan, Iran) and neomycin (500 mg neomycin sulfate per bolus; 2 boluses/d per calf before milk feedings for 5 consecutive days; Iran Pharmaceutical Products Co., Semnan, Iran). Once the aforementioned treatment was not effective, the animals received an intravenous liquid therapy using sodium bicarbonate solution (1.3%; 1.5 L/calf; Iran Pharmaceutical Products Co.), sugar and salt solution (dextrose 3.33% + sodium chloride 0.30%; 1 L/calf; Shahid Ghazi Pharmaceutical Co., Tabriz, Iran) with a single dose vitamin AD_3_E (containing 50000 IU vitamin A, 10000 IU vitamin D_3_, and 20 mg vitamin E per mL; 4 mL/calf; Rooyan Darou Co., Semnan, Iran) + B_12_-P complex (containing 0.05 mg cyanocobalamin and 125 mg sodium-α-oxybenzylphosphinicom per mL; 4 mL/calf; Razak Laboratories Co., Karaj, Iran) injection. Whenever a blood spot was seen in diarrhea, calves were treated with enrofloxacin (5%; 4 mL/calf for five consecutive days; Rooyan Darou Co.) and flunixin (5%; 4 mL per calf on the first day of treatment protocol; Razak Laboratories Co.) with a single dose of vitamin B_1_ (containing 200 mg thiamine hydrochloride per mL; 4 mL per calf on the first day of treatment protocol; Rooyan Darou Co.).

Regarding pneumonia, calves were administered with enrofloxacin (5%; 5 mL/calf for five consecutive days; Rooyan Darou Co.) and flunixin (5%; 4 mL per calf on the first day of the treatment protocol; Razak Laboratories Co.) with a single dose of multi-vitamin (containing 30 MIU vitamin A, 8 MIU vitamin D_3_, 16 KIU vitamin E, 2 g vitamin B_1_, 2 g vitamin B_2_, 20 g vitamin B_3_, 5 g vitamin B_5_, 2 g vitamin B_6_, 10 mg vitamin B_12_, 10 g vitamin C, 20 mg biotin, 30 g methionine, and 20 g lysine per L; 4 mL/d per calf on the first day of treatment protocol; Rooyan Darou Co.). Non-responding animals received this protocol again, plus Pantrisul (containing 200 mg trimethoprim and 200 mg sulfamethoxazole per mL; 5 mL per calf for 5 consecutive days; Makian Daru Co., Tehran, Iran).

### Statistical analyses

A pre-trial power analysis was performed to calculate the required number of calves for this study using ADG data as a reliable measure of calf growth from previously published papers [61,62]. Using PROC POWER in SAS (version 9.4; SAS Institute Inc., Cary, NC, USA) at 80% power (1 – β), half-widths at 0.05, a standard deviation of 100 g/d, and a minimum difference of 110 g/d for ADG among treatments, the sample size was calculated 51 calves totally (N = 17 per treatment). Data on nutrient intake, ADG, feed efficiency, growth performance (d 1 to d 101), sorting activity (d 51 to 101), and feeding behavior (pre- and post-weaning) were subjected to ANOVA using the mixed-effects model procedure of SAS with the period as repeated measures. The calf was considered as a random effect, and treatment (**T**; the effect of feeding WM, WM+MR, or WM+TM), period (**P**; 1- or 10-d period), and T × P interactions as fixed effects. For a pre-planned comparison of treatment means, *P*-values were specified for contrasts **C1** (WM *vs*. the average of WM+TM and WM+MR) and **C2** (WM+TM *vs*. WM+MR) using the Contrast statement of SAS. Data on initial BW and skeletal measurements were considered covariates for BW and skeletal growth. Several variance-covariance structures were tested, and the auto-regression structure (type 1) with minimized Bayesian information criterion was accordingly modeled. The effects of P and T × P were not reported in the tables for sorting activity and feeding behaviors because of non-significant P-values, however, they were modeled and analyzed. To determine whether sorting occurred, sorting activity for each fraction was tested for a difference from 100% using the *t*-test procedure in SAS. Data were reported as the least squares mean and considered significant if *P* ≤ 0.05; a tendency was reported if 0.05 < *P* ≤ 0.10.

Models for the occurrence of abnormal appearance (≥2), diarrhea (≥3), and pneumonia were evaluated independently before weaning (d 1 to 60) by logistic regression using a binomial distribution (0 or 1) in the GLIMMIX procedure of SAS. For example, a calf detected for abnormal appearance (≥2) was pointed by 1, while a calf with a good or normal appearance (<2) was pointed by 0. The same procedure was done for diarrhea (fecal score ≥3 = 1 and score <3 = 0) and pneumonia. The odds ratio was used to compare the likelihood of calves in each treatment group experiencing any event. In addition, the number of days with abnormal appearance (≥2), frequency and duration of diarrhea (≥3) or pneumonia, and administration of medication were tested by Poisson distribution using the GENMOD procedure of SAS.

The survival analysis model was performed using the Kaplan-Meier estimator of PROC LIFETEST in SAS, describing the probability (with a 95% confidence interval) of survival for the calves during the days of the experiment receiving the different milk supplements evaluated. To analyze the probability of survival or non-disease probability of abnormal appearance (≥2), diarrhea (≥3), or pneumonia using this model, data were distributed binomially (as followed for logistic model) as 1 (positive for disease or uncensored) or 0 (negative for disease or censored) for each variable independently. By this model, the non-disease probability of each variable was calculated from 1 (no risk) drop to 0 (completely at risk). We modeled survival as a function of calf age, with d 1 as the date of the calf’s birth up to weaning (60 days of life).

## Results

### Feed intake and growth performance

We observed no treatment effect on starter intake (SI) when the CON group was compared with the SUP animals (Table 3); however, animals fed MR-SUP had a superior SI than those fed TM-SUP (*P* = 0.006). When considering starter intake % of initial BW (SI_BW), animals in the CON presented a greater intake (*P* = 0.018); moreover, when evaluating the contrast of CON *vs*. SUP, animals receiving MR-SUP had a greater SI_BW than TM-SUP (*P* < 0.001). When considering the nutrient intake, calves in the SUP groups showed a greater dry matter intake (DMI) than those in the CON (*P* = 0.004); being the most significant results observed in the MR-SUP group with the highest DMI (*P* = 0.006). SUP groups showed a superior average BW (*P* = 0.037), and no effect was observed between the two types of supplements evaluated (*P* = 0.502). When evaluating ADG, the SUP groups presented a greater gain when compared with the CON group (*P* < 0.001), and no difference between SUP groups was observed in the C2 (*P* = 0.344); nonetheless, we observed that ADG in the function of metabolizable energy intake (ADG_ME) in the CON group showed a greater value when compared with the SUP groups (*P* = 0.001). Lastly, the CON group had a higher feed efficiency (FE) than SUP animals (*P* = 0.001), and between the SUP groups, TM-SUP was the one with the higher value (*P* = 0.003).

**Table 3:** Nutrient intake and growth performance as influenced by feeding pasteurized waste milk (WM) and a blend of WM with milk replacer powder (WM+MR) or transition milk (WM+TM) as liquid feeds to cold-stressed newborn Holstein calves (n = 17 per treatment).

Item^2^	Treatments (T)			SEM	Contrasts^1^		Fixed effects	
	WM	WM+MR	WM+TM		C1	C2	Period (P)	T × P
SI, g/d	1640.4	1668.0	1558.1	28.359	0.430	0.006	0.001	0.937
SI_BW, %	1.68	1.67	1.51	0.032	0.018	0.001	0.001	0.842
DMI, g/d	1984.8	2139.0	2028.6	28.411	0.004	0.006	0.001	0.962
DMI_BW, %	2.31	2.51	2.34	0.033	0.003	0.001	0.001	0.060
CPI, g/d	393.8	424.2	445.6	5.430	0.001	0.005	0.001	0.001
CFI, g/d	137.8	162.6	169.7	0.974	0.001	0.001	0.001	0.001
MEI, Mcal/d	6.67	7.35	7.14	0.086	0.001	0.075	0.001	0.345
Average BW, kg	79.19	80.29	80.79	0.530	0.037	0.502	0.001	0.999
ADG, kg/d	0.93	1.01	0.99	0.016	0.001	0.344	0.001	0.026
FE, kg/kg	0.62	0.51	0.57	0.013	0.001	0.003	0.001	0.001
ADG_ME, kg/Mcal	0.16	0.13	0.14	0.003	0.001	0.064	0.001	0.001

^1^Contrasts: C1 = WM *vs*. the average of WM+TM and WM+MR; C2 = WM+TM *vs*. WM+MR.

^2^Starter intake (SI); Starter intake % of initial body weight (SI_BW); Dry matter intake (DMI); Dry matter intake % of initial body weight (DMI_BW); Crude protein intake (CPI); Crude fat intake (CFI); Metabolizable energy intake (MEI); Average body weight (Average BW); Average daily gain (ADG); Feed efficiency (FE; kg of ADG/kg of DMI); and Average daily gain in the function of metabolizable energy intake (ADG_ME).

**Fig 1:**
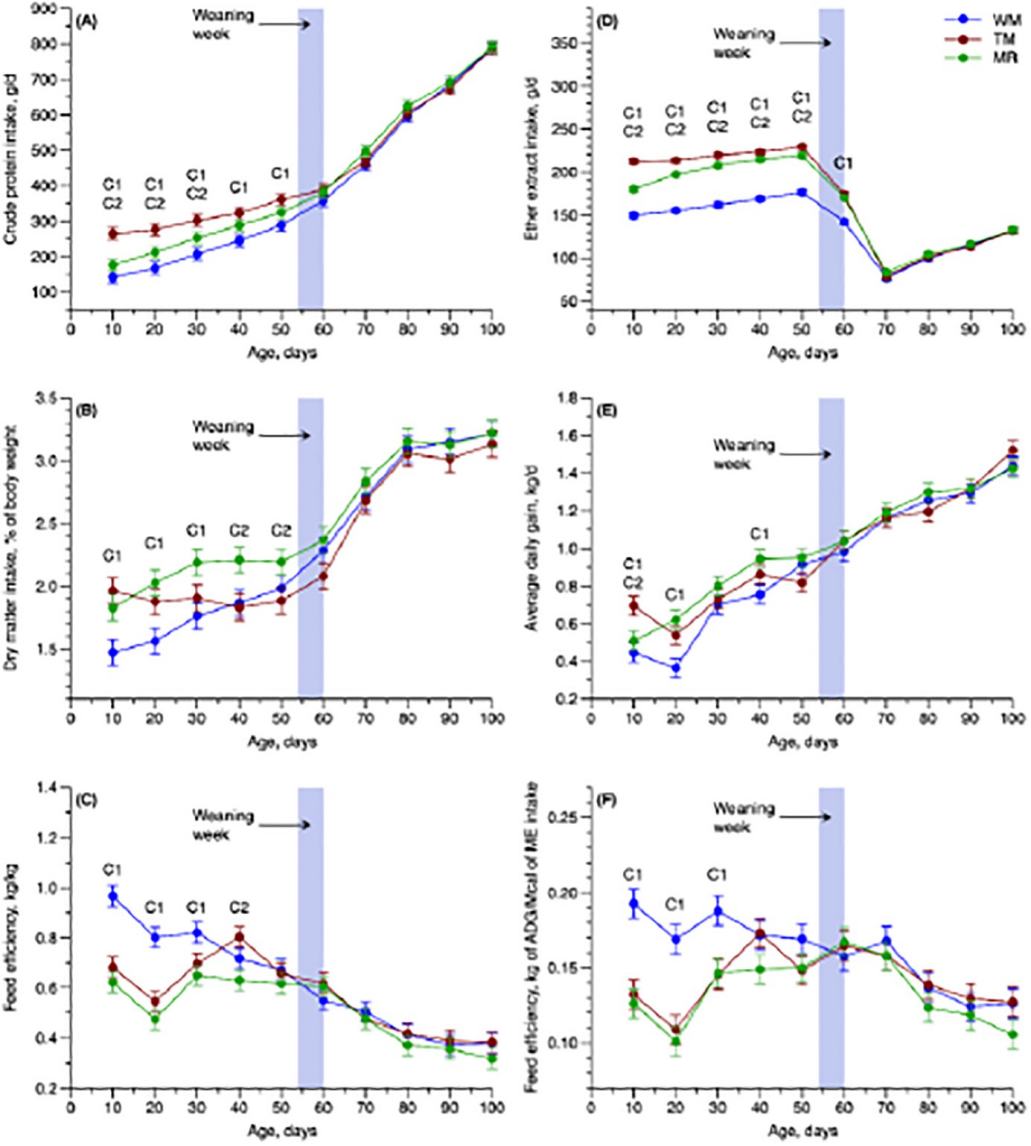
Interaction panel of treatment and period (T × P) for dry matter intake % of initial BW (DMI_BW, %), crude protein intake (CPI, g/d), crude fat intake (CFI, g/d), average daily gain (ADG, kg/d), feed efficiency (FE; kg of ADG/kg of DMI), and average daily gain in the function of metabolizable energy intake (ADG_ME, kg/Mcal) of the newborn calves receiving different sources of liquid feed. C1 (WM *vs*. WM+TM and WM+MR) and C2 (WM+TM *vs*. WM+MR) present the observed contrasts at each period. The wide gray column indicates the weaning week from day 54 to 60. Error bars represent SEM.

We observed an interaction effect of T × P for the following variables: crude protein intake (CPI; *P* = 0.001, Fig 1A), crude fat intake (CFI; *P* < 0.001, Fig 1D), ADG (*P* = 0.026, Fig 1E), FE (*P* < 0.001, Fig 1C), and ADG_ME (*P* < 0.001, Fig 1F). We also found a trend effect in the DMI_BW (*P* = 0.060, Fig 1B), but some significant differences were found within time points of preweaning. The supplementation increased the CPI and CFI of both SUP treatments through the preweaning phase, with a greater intake of animals fed TM-SUP up to 50 days of life (Fig 1A and 1D). The CON group showed a lower DMI_BW during the first 30 days of life, being greater DMI_BW in the preweaning phase found in the animals receiving MR-SUP (Fig 1B). The CON group showed the lowest ADG during the first 40 days of life, showing slight variations during the whole period of evaluation when compared with the SUP groups. Lastly, the CON group showed the greatest FE or ADG_ME in the first 30 days (Fig 1C and 1F).

**Fig 2:**
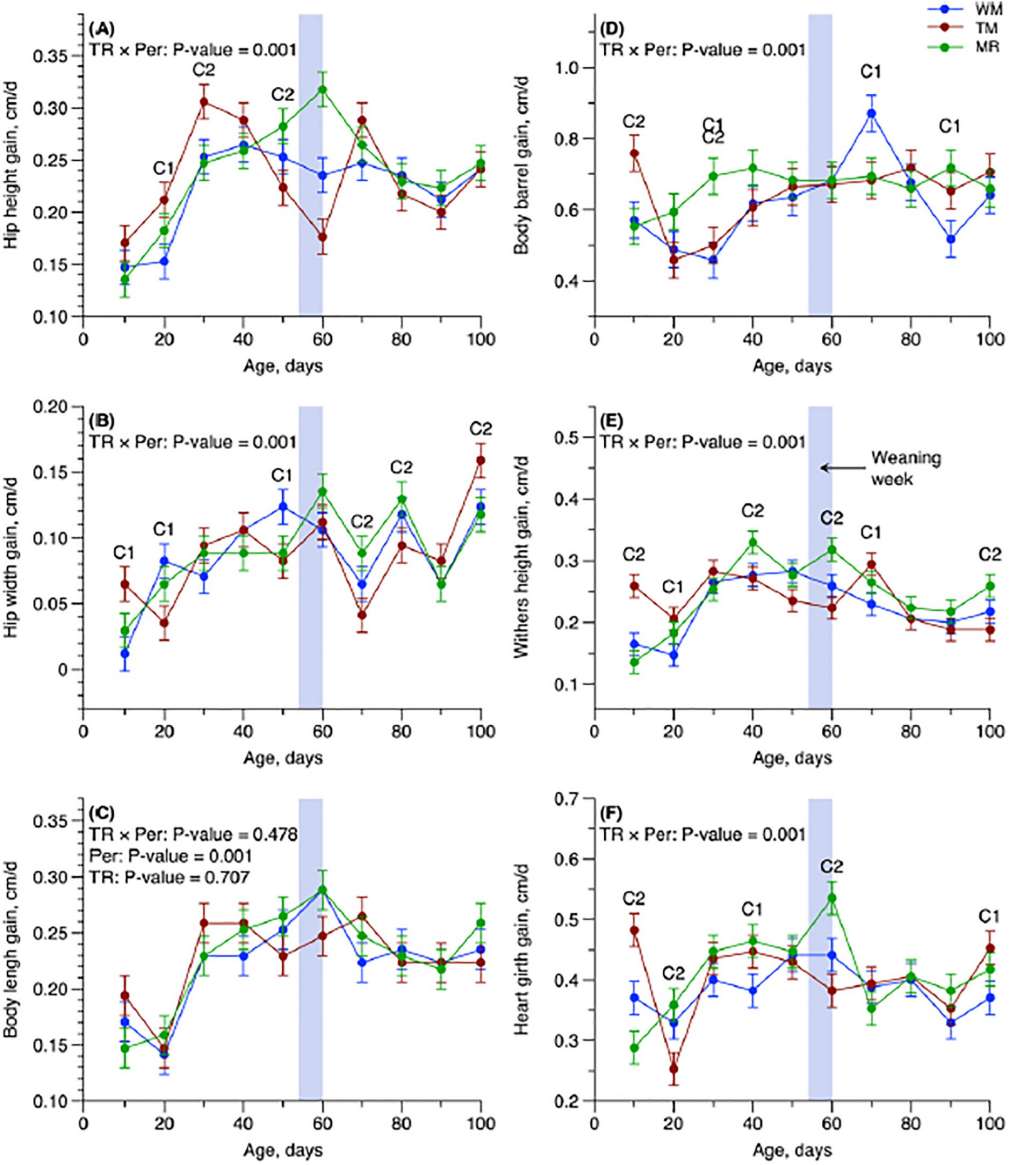
Interaction panel of treatment and period (T × P) for hip height gain (cm/d), hip width gain (cm/d), body barrel gain (cm/d), withers height gain (cm/d), and heart girth gain (cm/d) of the newborn calves receiving different sources of liquid feed. C1 (WM *vs*. WM+TM and WM+MR) and C2 (WM+TM *vs*. WM+MR) present the observed contrasts at each period. The wide gray column indicates the weaning week from day 54 to 60. Error bars represent SEM.

Animals in the SUP groups presented greater development on the skeletal growth parameters of withers height gain (*P* = 0.026) and heart girth gain (*P* = 0.042) when compared with the CON group (Table 4). The type of supplement (MR or TM) did not influence any of the growth parameters evaluated (Tables 3 and 4). However, some T × P interaction effects were found in the growth parameters data of hip height gain (*P* = 0.001, Fig 2A), hip width gain (*P* < 0.001, Fig 2B), body barrel gain (*P* = 0.001, Fig 2D), withers height gain (*P* = 0.001, Fig 2E), and heart girth gain (*P* = 0.001, Fig 2F).

**Table 4:** Skeletal growth as influenced by feeding pasteurized waste milk (WM) and a blend of WM with milk replacer powder (WM+MR) or transition milk (WM+TM) as liquid feeds to cold-stressed newborn Holstein calves (n = 17 per treatment).

Item	Treatments (T)			SEM	Contrasts^1^		Fixed effects	
	WM	WM+MR	WM+TM		C1	C2	Period (P)	T × P
Hip height gain, cm/d	0.22	0.24	0.23	0.005	0.075	0.384	0.001	0.001
Hip width gain, cm/d	0.09	0.09	0.09	0.004	0.816	0.687	0.001	0.001
Body length gain, cm/d	0.22	0.23	0.23	0.006	0.437	0.765	0.001	0.478
Body barrel gain, cm/d	0.62	0.67	0.64	0.016	0.057	0.302	0.001	0.001
Withers height gain, cm/d	0.22	0.25	0.24	0.006	0.026	0.197	0.001	0.001
Heart girth gain, cm/d	0.39	0.41	0.40	0.009	0.042	0.596	0.001	0.001

^1^Contrasts: C1 = WM *vs*. the average of WM+TM and WM+MR; C2 = WM+TM *vs*. WM+MR.

Overall, animals in the SUP groups exhibited superior body development compared with those in the CON group. Markedly, for the animals fed TM-SUP, there were remarkable skeletal measurements during the first 10 days of life. However, their performance was surpassed by those receiving MR-SUP across most of the variables analyzed throughout the 100-day evaluation period.

### Sorting behavior and feeding activity

Irrespective of the type of liquid feed fed, all calves sorted for particles retained on the 4.75-mm sieve during all studied periods (*P* ≤ 0.05); however, particles retained on the 2.36-mm sieve remained unsorted (Table 5). Furthermore, all animals sorted against particles retained on a 1.18-mm sieve; however, the extent of sorting was greater in MR- *vs*. TM-SUP calves (*P* = 0.038). Regardless of the type of liquid feed, all calves sorted against particles retained on the 0.6-mm sieve. Calves fed SUP groups sorted more for feed materials retained on the pan (*P* = 0.001) and the extent of sorting was greater in MR- *vs*. TM-SUP calves (*P* = 0.002).

**Table 5:** Sorting index^1^ as influenced by feeding pasteurized waste milk (WM) and a blend of WM with milk replacer powder (WM+MR) or transition milk (WM+TM) as liquid feeds to cold-stressed newborn Holstein calves (n = 17 per treatment).

Sorting index, %	Treatments			SEM	Contrasts^2^	
	WM	WM+MR	WM+TM		C1	C2
4.75 mm	113.3*	113.1*	112.6*	0.43	0.400	0.408
2.36 mm	100.2	100.0	99.8	0.34	0.592	0.639
1.18 mm	96.6*	96.1*	96.8*	0.23	0.521	0.038
0.6 mm	92.5*	92.5*	92.6*	0.35	0.899	0.825
Pan	100.8*	103.3*	101.9*	0.30	0.001	0.002

^1^Sorting % = 100 × (actual particle-size fraction DM intake/predicted particle-size fraction DM intake). Values equal to 100% indicate no sorting, <100% indicate selective refusals (sorting against), and >100% indicate preferential consumption (sorting for).

^2^Contrasts: C1 = WM *vs*. the average of WM+TM and WM+MR; C2 = WM+TM *vs*. WM+MR.

*P ≤ 0.05; sorting values differ from 100%.

Meal frequency, inter-meal interval, meal size, and starter feed intake (g/8 h period) were not affected by treatment; however, meal length and eating rate were greater (*P* = 0.05) and lower (*P* = 0.05) in calves fed SUP groups than those fed CON group, respectively (Table 6). Rumination frequency, rumination duration, and rumination interval were not affected by treatment. Time spent eating, ruminating, resting, drinking, and NNOB were not affected by treatment; however, calves in SUP groups had greater (*P* = 0.04) and lower (*P* = 0.04) standing- and laying times, respectively.

**Table 6:** Meal and rumination patterns, chewing behaviors (min/8 h), and times of resting, drinking, non-nutritive oral behaviors (NNOB), standing, and lying as influenced by feeding pasteurized waste milk (WM) and a blend of WM with milk replacer powder (WM+MR) or transition milk (WM+TM) as liquid feeds to cold-stressed newborn Holstein calves (n = 17 per treatment).

Item	Treatments			SEM	Contrasts^1^	
	WM	WM+MR	WM+TM		C1	C2
Meal						
Bouts (frequency)/8 h	5.1	5.2	5.0	0.30	0.67	0.80
Length, min	9.0	9.7	9.9	0.38	0.05	0.52
Interval, min	94.1	92.3	96.0	14.55	0.68	0.44
Eating rate, g of SI/min	21.1	19.6	19.0	0.90	0.05	0.46
Meal size, g of SI/bout	189.6	190.0	188.2	18.57	0.92	0.29
Starter DM intake, g/8 h	967.7	988.0	941.0	93.90	0.76	0.84
Rumination						
Bouts (frequency)/8 h	4.5	4.3	4.2	0.22	0.78	0.28
Length, min	18.2	18.2	18.3	0.66	0.99	0.93
Interval, min	106.7	111.6	114.3	10.30	0.84	0.26
Eating time, min	45.9	50.4	49.5	3.56	0.37	0.35
Ruminating time, min	81.9	78.3	76.9	4.24	0.59	0.26
Resting time, min	308.8	316.3	314.9	7.35	0.57	0.52
Drinking time, min	14.1	12.8	11.7	1.37	0.94	0.24
NNOB, min	29.3	22.2	27.0	3.18	0.13	0.62
Standing time, min	150.0	167.7	172.1	7.08	0.04	0.33
Laying time, min	330.0	312.3	307.9	7.08	0.04	0.33

^1^Contrasts: C1 = WM *vs*. the average of WM+TM and WM+MR; C2 = WM+TM *vs*. WM+MR.

### Health

Table 7 presents the logistic models for the occurrence of abnormal appearance (score ≥2), diarrhea (score ≥3), and pneumonia before weaning (d 1 to 60). The occurrences of abnormal appearance and pneumonia were lower (*P* ≤ 0.05) in SUP calves than in CON calves with no difference between MR- and TM-SUP. Calves in all treatment groups had a similar chance of having diarrhea.

**Table 7:** Logistic model for abnormal appearance (≥2)^1^, diarrhea (≥3)^2^, and pneumonia occurrences before weaning (d 1 to 60) as influenced by feeding pasteurized waste milk (WM) and a blend of WM with milk replacer powder (WM+MR) or transition milk (WM+TM) as liquid feeds to cold-stressed newborn Holstein calves (n = 17 per treatment).

Variable and comparison	Coefficient	SE	OR^3^	95% CI^4^	*P*-value
Abnormal appearance					
WM+MR *vs*. WM	−0.3303	0.154	0.71	0.53, 0.97	0.032
WM+TM *vs*. WM	−0.3718	0.156	0.69	0.50, 0.93	0.017
WM+MR *vs*. WM+TM	0.0414	0.166	1.04	0.75, 1.44	0.803
Diarrhea occurrence					
WM+MR *vs*. WM	−0.0986	0.134	0.90	0.69, 1.17	0.461
WM+TM *vs*. WM	−0.0986	0.134	0.90	0.69, 1.17	0.461
WM+MR *vs*. WM+TM	0.0000	0.136	1.00	0.76, 1.30	1.000
Pneumonia occurrence					
WM+MR *vs*. WM	−0.9068	0.398	0.40	0.18, 0.88	0.022
WM+TM *vs*. WM	−1.0255	0.415	0.35	0.15, 0.81	0.013
WM+MR *vs*. WM+TM	0.1188	0.487	1.12	0.43, 2.93	0.807

^1^1 = normal and alert; 2 = ears drooped; 3 = head and ears drooped, dull eyes, and slightly lethargic; 4 = head and ears drooped, dull eyes, and lethargic; and 5 = severely lethargic; based on the scoring system described by [56].

^2^1 = normal; 2 = soft to loose; 3 = loose to watery; 4 = watery, mucous, and slightly bloody; and 5 = watery, mucous, and bloody; based on the scoring system described by [56].

^3^The odds ratio (OR) indicates the probability of having abnormal or elevated general appearance (≥2), diarrhea (≥3) or pneumonia for the experimental treatments (WM+MR *vs*. WM; WM+TM *vs*. WM; and WM+MR *vs*. WM+TM). If the OR is >1, a given liquid feed in the comparison is more likely to have elevated general appearance, diarrhea, or pneumonia than the other liquid feed by a factor of the difference above 1. If the OR is <1, a given liquid feed has a lower probability of occurrence than the other liquid feed.

^4^Confidence interval.

Table 8 presents the Poisson regression for the frequency and number of days with abnormal appearance (score ≥2), diarrhea (score ≥3), pneumonia, and medicated days for both diarrhea and pneumonia. Calves in SUP groups experienced lower days (1.7 d) with abnormal appearance without difference between MR- and TM-SUP groups. Neither SUP nor type of balancer affected frequency, duration, and medication days for diarrhea. Frequency and medication days for pneumonia were not affected by treatment; however, calves in SUP groups experienced shorter days (0.8 d) with pneumonia, with no difference between MR- and TM-SUP calves.

**Table 8:** Poisson regression for days with abnormal appearance (≥2)^1^, frequency and duration of diarrhea (≥3)^2^ and pneumonia, and days with medication before weaning (d 1 to 60) as influenced by feeding pasteurized waste milk (WM) and a blend of WM with milk replacer powder (WM+MR) or transition milk (WM+TM) as liquid feeds to cold-stressed newborn Holstein calves (n = 17 per treatment).

Item	Treatments			SEM	Contrasts^3^	
	WM	WM+MR	WM+TM		C1	C2
Abnormal appearance, d	6.35	4.71	4.53	0.107	0.022	0.326
Diarrhea						
Frequency, no of times	1.41	1.47	1.29	0.205	0.768	0.735
Duration, d	7.76	7.18	7.18	0.089	0.530	0.720
Medication, d	5.88	5.59	5.88	0.101	0.998	0.679
Pneumonia						
Frequency, no. of times	0.29	0.24	0.24	0.482	0.738	0.852
Duration, d	1.29	0.53	0.47	0.299	0.009	0.310
Medication, d	1.47	1.18	1.18	0.215	0.455	0.676

^1^1 = normal and alert; 2 = ears drooped; 3 = head and ears drooped, dull eyes, and slightly lethargic; 4 = head and ears drooped, dull eyes, and lethargic; and 5 = severely lethargic; based on the scoring system described by [56].

^2^1 = normal; 2 = soft to loose; 3 = loose to watery; 4 = watery, mucous, and slightly bloody; and 5 = watery, mucous, and bloody; based on the scoring system described by [56].

^3^Contrasts: C1 = WM *vs*. the average of WM+TM and WM+MR; C2 = WM+TM *vs*. WM+MR.

**Fig 3:**
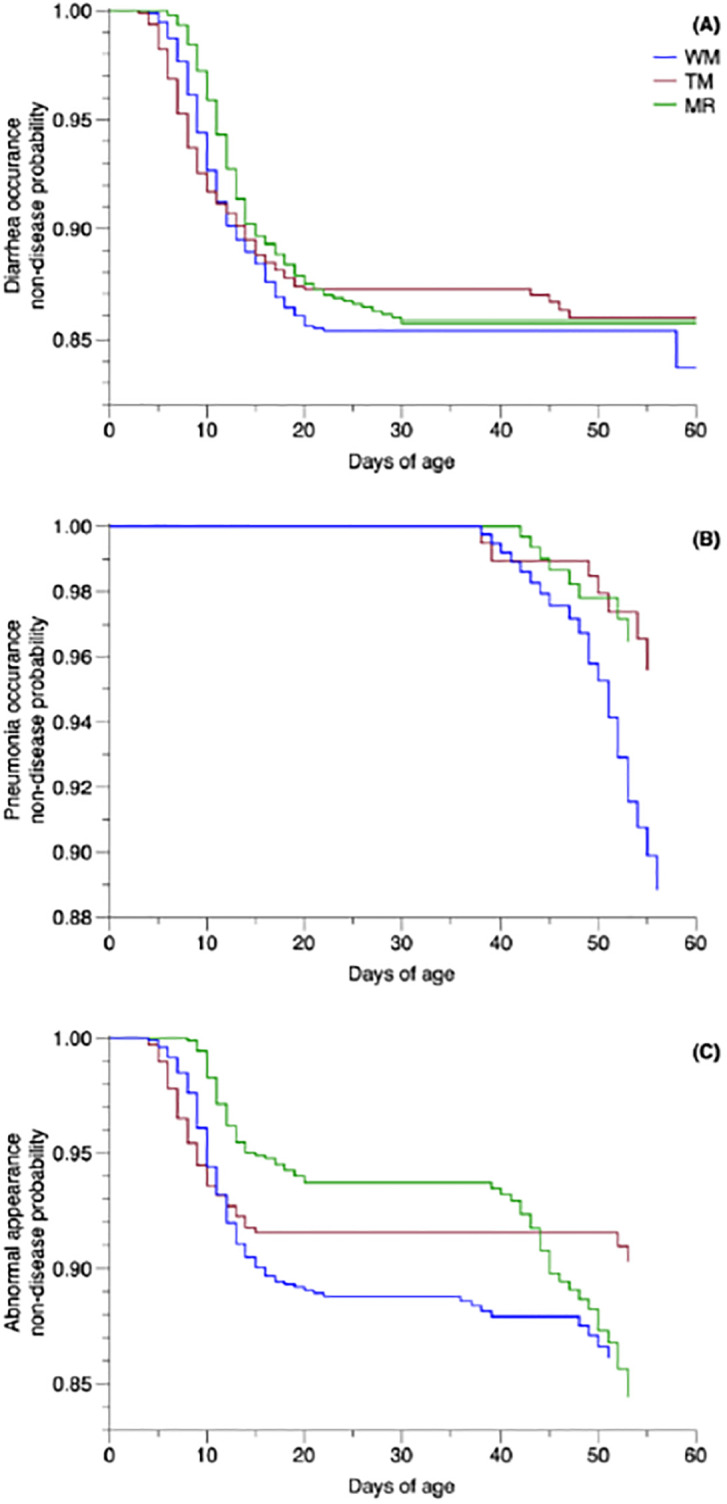
Survival function by Kaplan-Meier estimator for the non-disease probability of diarrhea or pneumonia and abnormal appearance before weaning (d 1 to 60) in the calves receiving different sources of liquid feed.

When evaluating the survival analysis by Kaplan-Meier curves for health data, we observed no significant effect of treatment on the occurrence of diarrhea (*P* = 0.693). However, it is possible to observe that the decrease in the probability of diarrhea incidence was more pronounced until 20 days of life for CON and TM-SUP groups, and after that point, the probability was stable until weaning. Animals in the TM-SUP group exhibited declines in non-disease probability beyond the initial 40 days. In the MR-SUP, this decrease was observed between the 20th and 30th days, whereas the CON group displayed a further reduction in the proximity of the weaning date (Fig 3A). Furthermore, both pneumonia occurrence and abnormal appearance were significant for the SUP groups (*P* = 0.012 and *P* = 0.027, respectively). In addition, we observed that calves from the CON group showed reduced health status when compared with the SUP groups. In terms of pneumonia occurrence (Fig 3B), the non-disease probability was consistent across all treatments until the animals reached 40 days of age. Subsequently, a decreasing trend was observed across all groups leading up to the weaning phase. Notably, the most notable decline was evident in the WM group, trailed by the TM- and MR-SUP groups.

## Discussion

The thermoneutral comfort zone of young calves experience variations due to factors such as age, weight, environmental conditions, and other stressors in the preweaning phase. Young calves, especially neonates, are highly susceptible to heat loss due to their high surface area-to-body mass ratio, lesser body fat, and underdeveloped thermoregulatory mechanisms compared to older calves [63]. As a result, when temperatures stray from this optimal range, it can impact calf behavior and physiology in various ways. These include increased energy expenditure to maintain warmth, decreased activity levels to minimize heat loss, the instinct to seek shelter, and the tendency to huddle together with other calves for warmth [64,65]. All those changes can potentially impact growth rates and overall health [66].

**Fig 4:**
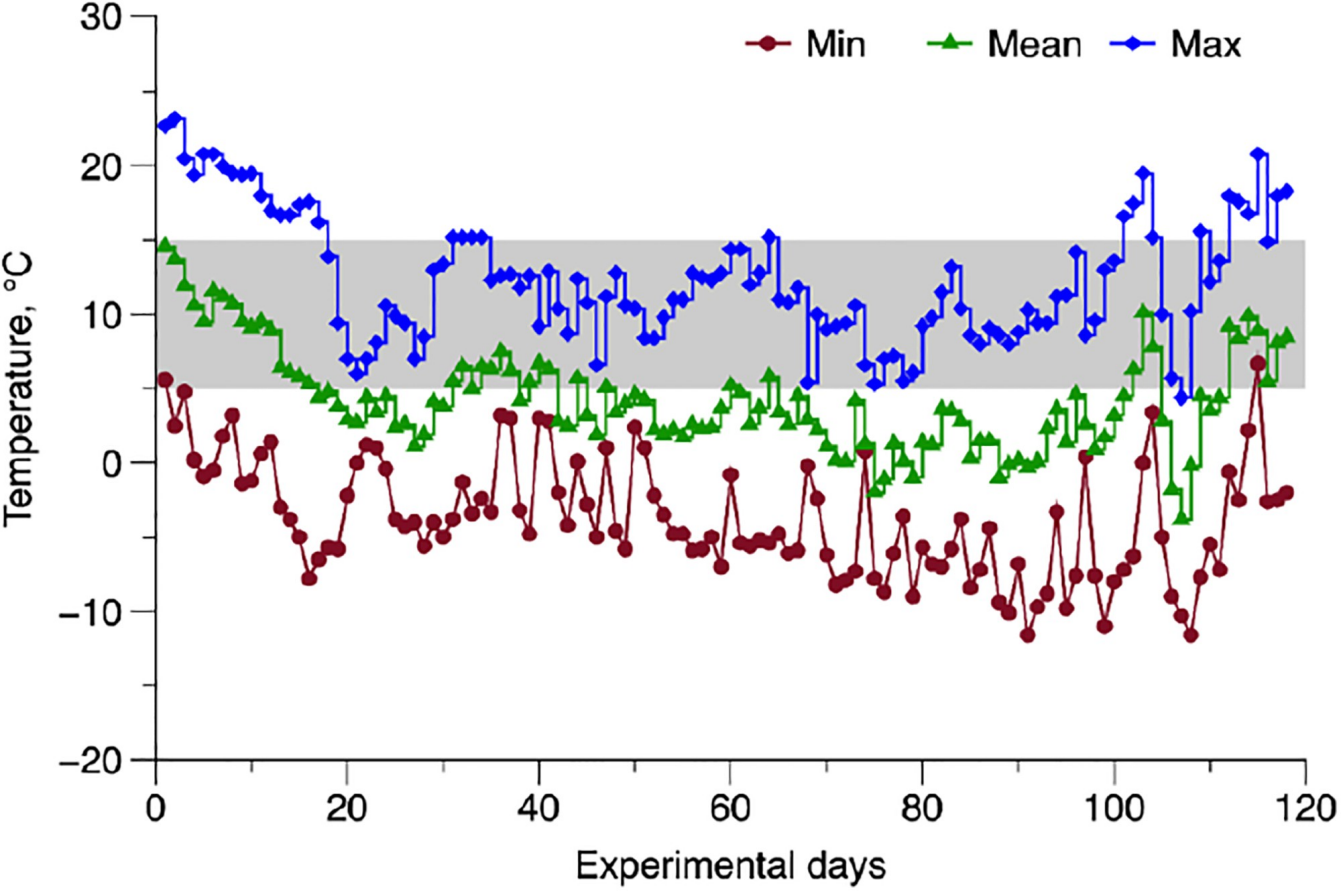
Temporal pattern of minimum (Min), mean, and maximum (Max) temperature during the experiment. The average minimum, mean, and maximum temperatures observed during the trial conduction were -3.8, 4.2, and 12.4 °C, respectively. The gray area indicates the lower critical temperature of 15 or 5 °C for newborn calves [16,17].

Throughout most of the study period, the average temperature remained around 5 °C (refer to Fig 4). This temperature marks a significantly lower threshold for both newborns (15 °C [16]) and calves older than 21 days (5 °C [17]). When the ambient temperature reaches 5 °C, the maintenance energy requirements show a 40% increase for calves up to 3 weeks old and a 13% increase for calves older than 3 weeks old [49], as their high surface area-to-volume ratio demands extra energy to sustain body heat, compounded by shivering thermogenesis and immature digestive systems hinder efficient energy extraction from feed, further elevating energy needs to meet maintenance requirements in cold temperatures [11,63,67]. As a result, it is crucial to adjust the animals’ diet to account for these changes with either energy-dense milk replacers or starter feeds. This dietary adjustment becomes essential to enable the calves to effectively manage cold stress, thereby preventing any compromise to their performance and health. Thus, the supplementation of WM-fed calves with TM or MR emerges as a potential solution to ensure producers that calves are receiving optimal nutrition, especially in situations where WM quality or quantity may be insufficient. However, there are risks associated with this practice. Overfeeding can lead to digestive issues, such as diarrhea, as well as metabolic disorders due to excessive intake of certain nutrients. Additionally, improper mixing or storage of supplemented milk can compromise its quality and increase the risk of bacterial contamination. Factors to consider include the nutritional composition of both WM and the supplement, the specific needs of the calf, and the management practices implemented to ensure proper feeding protocols are followed [34,35,36]. Regular monitoring of calf health and growth, along with consultation with a veterinarian or nutritionist, is essential to mitigate risks and optimize the benefits of supplementation with TM or MR. Being all considered, this supplementation could play a vital role in increasing the overall solids intake for neonates, helping them cope with the stress resulting from low ambient temperatures during their initial weeks of life.

The lower performance observed in the CON group can be attributed to the climatic conditions under which the animals were raised, coupled with a less dense diet in total solids. It is well-established that restrictive feeding has a negative impact on cold tolerance, particularly in young calves, as their body fat reserves are quickly depleted [18,49]. Considering this, enhancing the total solids content in a diet for calves experiencing cold stress, such as through the addition of TM or MR powder to WM, proves beneficial for the animals’ performance and intake, particularly during the initial 30 days of life (Table 3 and Fig 1). A study showed that pre-ruminant calves raised in the cold environment (mean temperature 5 °C) had similar weight gains in comparison with those raised in the warm environment (mean temperature 15 °C), although they consumed more solid feed [4].

CPI and CFI exhibited superiority within the TM-SUP group, potentially attributed to the chemical composition featuring higher levels of CP and CF in contrast to WM and WM+MR (as outlined in Table 1). Additionally, it is worth noting the significant role that TM plays in the early nutrition of newborn calves. Conceptually, it is the milk produced in the days following colostrum and preceding mature milk, while it represents a pivotal role in providing essential nutrients for the growth and development of newborn calves [68], it also presents potential benefits and drawbacks. On the positive side, its unique milk composition, characterized by elevated levels of proteins, fats, carbohydrates, vitamins, minerals, and growth factors, facilitates the transition from passive immunity provided by colostrum to the nutritional support required for the development of the calf’s gastrointestinal tract and immune system, facilitating adaptation to a diet of solid feed [69,70]. However, variations in TM composition, influenced by factors such as lactation stage, cow health, and environmental conditions, may pose challenges for calf nutrition and management [71]. Despite observing that animals receiving MR-SUP had the highest DMI among the assessed groups, it’s noteworthy that MR is commonly utilized for calf nutrition due to its various merits, including cost-effectiveness, biosecurity, and consistency compared with other non-saleable milk types like TM or WM. An investigation of heifer-raising operations in the United States indicated that 86% of surveyed farmers incorporated some form of MR, with 68% exclusively using MR for calf rearing [72]. A separate study focusing on crossbred Holstein-Gyr heifers revealed that elevating the total solids content in a liquid feed to 20.4% resulted in improved performance and body frame development during the pre- and post-weaning phases of dairy heifers. Remarkably, this modification failed to impact the consumption of solid feed or overall health. However, our study possibly diverged from these findings, potentially because our diets contained a lower total solids concentration of 14% compared with the concentration of 20.4% [42].

When assessing the interaction panel, several observations can be made. In both C1 (CON *vs*. TM- and MR-SUP) and C2 (TM-SUP *vs*. MR-SUP) interactions, the DMI of animals fed MR-SUP was superior to the CON group and within the SUP groups. This can likely be attributed to the feeding consistency associated with the MR formulation and mixing, which is particularly important for young animals. Consistency of feeding a liquid diet has been shown to support better gain rates in pre-weaned calves [40]. However, our study observed that animals fed only WM performed worse compared with the SUP groups.

Feeding variable amounts of MR to calves under stressful conditions can significantly impact calf performance, potentially leading to increased disease incidence and mortality rates. Findings in the present study demonstrated that calves fed variable amounts of MR, despite being fed higher nutrient levels compared with a CON group, performed poorly under stressful conditions [73]. Feeding additional MR mostly increased disease incidence and led to a trend for greater preweaning mortality in calves fed larger MR amounts. The authors have observed that additional MR feeding resulted in greater health problems, with diarrhea, electrolytes treatment, and antibiotics therapy [73]. Despite being a convenient, safe, consistent, and cost-effective source of nutrients for dairy calves [58], the impact of MR feeding on post-weaning ADG remains inconclusive, particularly at higher feeding rates [74–77]. Inconsistencies in MR feeding can result in nutritional stress, disrupting the development and function of the gastrointestinal tract and weakening the immune system. To mitigate these risks, ensuring consistent and adequate nutrition through regular MR feeding is crucial. Implementing proper management practices and closely monitoring calf health can help minimize the negative impacts of variable MR feeding and improve overall calf performance and welfare under stressful conditions [78,79].

Studies assessing body development have shown a decrease in body traits during post-weaning evaluation [75,80]. Intensive MR feeding programs have been associated with a slowdown in the digestion of post-weaning diets consisting solely of solid feed, preventing the full absorption and utilization of nutrients for growth and development [74,81]. Consequently, the decline in the rate of digestion of solid feed may be responsible for the decreased ADG and structural growth observed in calves receiving MR feeding programs [82].

The observed trends in our study revealed that the calves of SUP groups exhibited superior MEI, while the CON group displayed higher ADG_ME. Two distinct explanations emerge for these trends, each aligned with a specific comparison: 1) when examining the contrast between the CON and SUP groups, we postulate that the developmental limitations of the calves could have been magnified by cold ambient temperatures. In cold conditions, the inability of increased nutrient intake, such as energy, to keep pace with heightened heat production leads to reduced performance [11] and elevated morbidity and early-life mortality [10]. A survival analysis evaluation, which considered the most prevalent health issues in calf rearing, namely diarrhea, and pneumonia, along with an assessment of abnormal appearances in the animals, was conducted. These health challenges result from a complex interaction of factors, including feeding practices, climate, and housing management [83]. Our findings pointed to the CON group calves having more severe pneumonia-related health conditions and abnormal appearances (Fig 3B and 3C). Additionally, the harsh cold conditions can compromise young calves’ health status and immune function, like the impact observed in animals raised in hot conditions, thereby enhancing susceptibility to diseases during the preweaning phase [44]. Considering these factors, we conclude that the supplementation with either TM or MR played a vital role in enabling cold-stressed calves to effectively manage adverse weather conditions. 2) Shifting our focus to the comparison within the SUP groups (MR-SUP *vs*. TM-SUP), differences in the nutritional composition of TM or MR likely influenced distinct growth patterns in each calf subgroup. Manipulating the protein-to-energy ratio in MR can noticeably reshape the body composition of preruminant calves. The concentration of fat in empty-body gain exhibited an inverse relationship with that of water and protein as dietary CP concentration escalated [84]. Despite TM having the highest CP to ME ratio (g/Mcal), this advantage failed to translate into observable performance gains (as depicted in Table 3). We anticipated that a higher CP to ME ratio in TM would yield better performance than MR [85]. Calves from the SUP groups exhibited elevated body measures (ADG, body barrel, withers height, and heart girth), which might be attributed to the greater intake of SI and DMI, possibly contributing to more substantial overall body development. In a broader assessment, it’s evident that animals within the SUP groups demonstrated heightened growth during both pre- and post-weaning periods compared with those within the CON group.

In summary, we observed an interaction between the supplementation and intake of newborn calves, mainly regarding dry matter intake, crude protein, and crude fat over the preweaning period. The observed increase in total solids feeding serves as an alternative to assist calves in coping with cold ambient temperatures in the first weeks of life. These changes were followed by improved performance during the first 40 days of life, with better overall body development, a decrease in the incidence of pneumonia in the preweaning phase, and improved general health appearance of the calves that received supplementation regardless of the type of milk used.

## Conclusion

We concluded that both TM and MR supplementation played key roles in enabling calves to cope better with the challenges of cold stress, with minimal differences between them. However, considering the main growth patterns, performance, and health metrics, our findings suggest that TM supplementation emerged as a particularly beneficial strategy, with implications for both pre- and post-weaning periods, turning out as a promising avenue for enhancing calf growth and well-being, especially in the face of challenging weather conditions.
